# Editorial: Clinical Translation and Commercialisation of Advanced Therapy Medicinal Products

**DOI:** 10.3389/fbioe.2020.619698

**Published:** 2020-12-09

**Authors:** Ivan Martin, Yves Bayon, Tracy T. L. Yu, Alain A. Vertès

**Affiliations:** ^1^Department of Biomedicine, University Hospital Basel, University of Basel, Basel, Switzerland; ^2^Department of Biomedical Engineering, University of Basel, Basel, Switzerland; ^3^Medtronic – Sofradim Production, Trévoux, France; ^4^M Ventures, Amsterdam, Netherlands; ^5^NxR Biotechnologies GmbH, Basel, Switzerland

**Keywords:** advanced therapy medicinal products (ATMPs), clinical translation, cell therapy, gene therapy, market access, cell manufacturing, tissue engineering, regenerative medicine

In the past 5 years, the number of ongoing clinical trials in advanced therapy medicinal products (ATMPs) worldwide has been increasing tremendously, at a staggering compounded annual growth rate (CAGR) of 14% (from 631 in 2015 to 1066 in 2019, source: Alliance for Regenerative Medicine). The field as a whole, though still emerging, has attracted on average $10 billion of financings every year, with the Gene & Gene-modified Cell Therapy category leading the way, accounting for more than half of the total financings toward all ATMPs. This funding injection among other factors has fuelled the growth in the number of companies created in this space, at a worldwide CAGR of 10.1% (2015-2019; source: Alliance for Regenerative Medicine). Geographically, Asia emerges as the fastest growing region in the number of companies enabled not only by foreign direct investments but also by national investments, followed by North America and Oceania (CAGR from 2015 to 2019: 12.6%, 11.2%, and 10%, respectively; source: Alliance for Regenerative Medicine). Remarkably, Asian countries, and notably Japan amongst the very first, have adapted their regulatory processes to implement faster these transformational therapies in areas of high unmet needs. Nonetheless, there still remains several critical hurdles before the full realization of the therapeutic, market, and economic potential of ATMPs, including: (1) remaining perceived technology risk; (2) still slow rate of overall technology adoption; (3) still limited market access complexified by business model challenges particularly when it comes to autologous products and exacerbated by previously unexplored pricing and reimbursement issues; and finally (4) still limited overall manufacturing capacity compared to the fast increasing demand from numerous clinical trials and from the market itself post novel ATMPs' product launch. On the other hand, there are key enabling factors including: investments; policies and notably advanced regulatory policies; Phase III data readouts and product approvals; as well as tailor-designed business ecosystems adapted to the intrinsic characteristics of cell and gene-based therapies. These four dimensions constitute important catalysts to accelerate the pace of the development of this sector. Currently, more than 25 ATMPs are available in the market such as Alofisel (2018), LUXTURNA® (2017), YESCARTA® (2017), KYMRIAH® (2017), and INVOSSA™ (2017).

When tackling this Research Topic, we aimed to provide diverse perspectives and learnings on the clinical translation and commercialization front of the ATMP field, as well as to feature several biotechnologically significant original research work. Ghamari et al. highlighted the current discrepancies in the marker characterization of placenta-derived amniotic cells with the aim of achieving better clinical translation and safer practices. Similarly, in an original research article, Asnaghi et al. exemplified the importance of biomarker signatures for the quality of engineered nasal chondrocyte-derived cartilage, where they proposed gene expression patterns and generalized linear models could be used to define the molecular signatures of identity, purity, and potency of regenerative cellular therapies. In addition, in another original research article, Rothweiler et al. proposed a novel predictive MSC chondrogenesis marker, the ratio of TGFβ-RI/TGFβ-RII, which could be tuned by siRNA knockdown of TGFβ-RII and subsequently recover the chondrogenic differentiation ability of non-responsive MSCs.

As alternatives to autologous/allogenic cell therapy approaches, cell-derived extracellular vesicles, and acellular xenografts could bring unique opportunities, at the same time associated with distinct challenges. In a review article, Maumus et al. brought forward some of the key aspects in production, regulation, and clinical translation, while He et al. presented a pre-clinical study of acellular xenograft from whole porcine meniscus as a potential substitute to partially replace irreparable damaged meniscus.

Looking at ATMP applications by tissue types, Sallent et al. reviewed and discussed the clinical translation and regulatory aspects of bone grafts by identifying the patterns of successful clinical translations, which are related to the understanding of the mechanism of action of the various device components and their compliance with regulatory frameworks. In another review, Magrelli et al. analyzed the ophthalmology field as an example of the health economics of ATMPs in comparison with conventional therapies, coming to the interesting conclusion that these two therapeutic approaches are actually economically comparable.

Needless to say, it is impossible to have a complete Research Topic on ATMPs without considering cell manufacturing and its delivery logistics. The former was featured in a mini review article by Doulgkeroglou et al., in which they proposed that it is critical to consider automation, monitoring, digital tools and standardization of cell product manufacturing at the early phase of the product development cycle. The latter was exemplified by a cell therapy logistic tool innovation that has the potential to offer a novel portable solution for live cell transportation by Willbrand et al..

A highlight of this Research Topic is from the remarkable translation of an allogenic cell product—primary dermal progenitor fibroblasts (FE002-SK2 cell type). In this original research article, Laurent et al. reported a success story and learnings ranging from the mechanistic characterization, bioprocessing up-scaling, and all the way to cross-continental technology and material transfer.

The field of ATMPs is likely at or nearing its inflection point, thus announcing tremendous changes to come and a further acceleration in discoveries, development, and translation to the bed-side, which will profoundly transform healthcare in disease areas and indications that have all but vexed the pharmaceutical and biotechnological industry to this date. Transformational changes are desperately needed in numerous indications with high unmet need, such as heart diseases, neurodegenerative diseases, fibroses of the liver or kidney, solid tumours, and not to forget tissue and organ replacement, where conventional therapeutic modalities have failed to this date to provide a suitable solution. However, there are multifarious challenges that must be overcome to reach clinical fruition for a large number of patients. We trust that this Research Topic highlights relevant readings to capture the current status and the potential for the development of ATMPs.

The previous decade was that of reaching a generally recognized proof of concept for cell and gene-base therapies. This coming decade is that of implementing in a large number of indications the promises of the novel therapies. Such promises are best exemplified by Provenge, Glybera, KYMRIAH®, and Yescarta®. These products all constitute critical precursor products regardless of their past, present, or future commercial successes. The first approvals of cell and gene therapies in key jurisdictions have paved the way for a transformation of healthcare as radical as the one triggered by the technology of monoclonal antibodies. Successive innovations, market access, and advanced manufacturing will increasingly play a central role as the first technology wave matures. They should enable, in a domino effect, the emergence of the next generation ATMPs, such as: (1) living drugs that are engineered to a higher degree of complexity to permit better therapeutic function in terms of both safety and efficacy, (2) novel cell types (e.g., macrophages) that harness unspoiled yet fundamental biological properties, and (3) novel combination therapies to exploit synergies between conventional and advanced therapies to deliver greatly optimized treatment algorithms to patients with life-threatening diseases.

## Author Contributions

All authors listed have made a substantial, direct and intellectual contribution to the work, and approved it for publication.

## Conflict of Interest

YB is an employee of Sofradim Production, A Medtronic Company. TY is employed by M Ventures, the corporate venture capital fund of Merck KGaA. AV is the Managing Director of NxR Biotechnologies GmbH. The remaining author declares that the research was conducted in the absence of any commercial or financial relationships that could be construed as a potential conflict of interest.

